# Resolving Individuals Contributing Trace Amounts of DNA to Highly Complex Mixtures Using High-Density SNP Genotyping Microarrays

**DOI:** 10.1371/journal.pgen.1000167

**Published:** 2008-08-29

**Authors:** Nils Homer, Szabolcs Szelinger, Margot Redman, David Duggan, Waibhav Tembe, Jill Muehling, John V. Pearson, Dietrich A. Stephan, Stanley F. Nelson, David W. Craig

**Affiliations:** 1 Translational Genomics Research Institute (TGen), Phoenix, Arizona, United States of America; 2 University of California Los Angeles, Los Angeles, California, United States of America; Queensland Institute of Medical Research, Australia

## Abstract

We use high-density single nucleotide polymorphism (SNP) genotyping microarrays to demonstrate the ability to accurately and robustly determine whether individuals are in a complex genomic DNA mixture. We first develop a theoretical framework for detecting an individual's presence within a mixture, then show, through simulations, the limits associated with our method, and finally demonstrate experimentally the identification of the presence of genomic DNA of specific individuals within a series of highly complex genomic mixtures, including mixtures where an individual contributes less than 0.1% of the total genomic DNA. These findings shift the perceived utility of SNPs for identifying individual trace contributors within a forensics mixture, and suggest future research efforts into assessing the viability of previously sub-optimal DNA sources due to sample contamination. These findings also suggest that composite statistics across cohorts, such as allele frequency or genotype counts, do not mask identity within genome-wide association studies. The implications of these findings are discussed.

## Author Summary

In this report we describe a framework for accurately and robustly resolving whether individuals are in a complex genomic DNA mixture using high-density single nucleotide polymorphism (SNP) genotyping microarrays. We develop a theoretical framework for detecting an individual's presence within a mixture, show its limits through simulation, and finally demonstrate experimentally the identification of the presence of genomic DNA of individuals within a series of highly complex genomic mixtures. Our approaches demonstrate straightforward identification of trace amounts (<1%) of DNA from an individual contributor within a complex mixture. We show how probe-intensity analysis of high-density SNP data can be used, even given the experimental noise of a microarray. We discuss the implications of these findings in two fields: forensics and genome-wide association (GWA) genetic studies. Within forensics, resolving whether an individual is contributing trace amounts of genomic DNA to a complex mixture is a tremendous challenge. Within GWA studies, there is a considerable push to make experimental data publicly available so that the data can be combined with other studies. Our findings show that such an approach does not completely conceal identity, since it is straightforward to assess the probability that a person or relative participated in a GWA study.

## Introduction

Resolving whether an individual's genomic DNA is present at trace amounts within a complex mixture containing DNA from numerous individuals is of interest to multiple fields. Within forensics, determining whether a person contributed their DNA to a mixture is typically a manual process that requires extensive experience and careful training. Furthermore, different laboratories can often come to different conclusions due to differences in methodology or lab intervariability. In large part, forensically identifying whether a person is contributing less than 10% of the total genomic DNA to a mixture is not easily done, is difficult to automate, and is highly confounded with the inclusion of more individuals. Within the field of forensics, as well as the field of human genetics, there is a base assumption that it is not possible to identify individuals using pooled data (e.g. allele frequency) from SNP data. In this paper we investigate the accuracy of such assumptions.

Numerous methods examining DNA mixtures currently exist, most of these addressing mixtures with smaller numbers of individuals within forensics studies [1]–[3]. Using short tandem repeats (STR) is a common method to generate DNA genotyping profiles and allows for identification of the various alleles and their relative quantity within the mixture [4]–[7]. Frequently, STRs on the Y chromosome are useful when resolving the male components of the mixture [8]. Nevertheless, these methods based on STRs expectedly suffer from limited power when using severely degraded DNA [8],[9]. Mitochondrial DNA (mtDNA) based on hypervariable region sequencing is useful when analyzing degraded DNA due to its high copy number and improved stability. Profiles for mtDNA can also be combined with STR analysis for better identification [10]. Nonetheless, mtDNA has weaknesses, including the uniparental mode of inheritance and lower discrimination power that can be moderately mediated by using the whole mitochondrial genome or known surrounding single nucleotide polymorphisms (SNPs) [11],[12]. Informative SNPs have been used to help resolve problems with using mtDNA [11],[13],[14] but have not been used wholly or separately as the discriminatory factor, or on the same scale as we propose.

In this study, we assess the feasibility of using hundreds of thousands of SNPs assayed on a high-density microarray as a means to resolve trace contributions of DNA to a complex mixture. High-density SNP genotyping arrays have predominately been developed as tools for geneticists to identify common genetic variants that predispose an individual to disease. In the context of forensic mixtures, SNPs are traditionally analyzed by genotype (e.g. AA, AT, or TT) and thought to be non-ideal for resolving mixtures. In fact, it is argued that their poor performance in the analysis of mixed DNA samples is one of the primary reasons SNP genotyping arrays have not become adopted by the forensics community [8],[15]. However, most SNP assays are inherently quantitative at one or both alleles, requiring a genotype calling algorithm to digitize the inherently analog information of a SNP assay [16]. Within this paper, we specifically exploit raw allele intensity measures for analysis of DNA with mixed samples.

We demonstrate an approach for rapidly and sensitively determining whether a trace amount (<1%) of genomic DNA from an individual is present within a complex DNA mixture. We focus on solving the problem forensically, whereby the problem is much more difficult due to the multiple sources of experimental noise that would further mask identification. Our method can be interpreted as a cumulative analysis of shifts in allele probe intensities in the direction of the individual's genotype. Similarly, we can also interpret our method as measuring the difference of two distances: the distance of the individual from a reference population and the distance of an individual from the mixture. Our method does not require knowledge of the number of individuals in the mixture and we demonstrate robustness for discriminating mixtures composed of over a thousand individuals. We first give a theoretical justification for our method with modifications for known factors including homogeneity of the mixture and accuracy of our reference populations. We then proceed to simulate the effects of three combinations of variables when using SNP microarrays, including probe measurement noise, fraction of the person of interest's DNA in the mixture, and the number of SNPs probed. Finally in a series of proof-of-principle experiments using both Affymetrix and Illumina microarrays, we demonstrate resolving whether an individual is within a series of complex mixtures (2 to 200 individuals) when the individual contributes trace levels (at and below 1%) of the total genomic DNA. We finally discuss the implications of these results in the context of forensics and population genetics.

## Methods

### Complex Mixture Constructions

A total of 8 complex mixtures were constructed (See Table 1). All DNA samples were checked for concentration in triplicates using the Quant-iT PicoGreen dsDNA Assay Kit by Invitrogen (Carlsbad, CA). For accuracy, an eight point standard curve was prepared using Human Genomic DNA from Roche Diagnostics (Cat#: 11691112001, Indianapolis, IN). The median concentrations were calculated for each individual DNA sample.

**Table 1:** Mixtures are composed partially of HapMap individuals empirically evaluated on the Illumina 550 K v3, Illumina 450S Duo, and Affymetrix 5.0 microarrays.

Name	Description	Illumina		Affymetrix
		550 K	450S	5.0
Mixture A	**Equimolar pool.** Equimolar mixture of 41 CEU individuals (14 Trios minus one individual)	Yes	No	Yes
Mixture B	**Equimolar pool.** Equimolar mixture of 47 CEU individuals (16 Trios minus one individual)	Yes	No	Yes
Mixture C	**2-person mixture.** 90% one CEU individual, 10% a second CEU individual	Yes	No	Yes
Mixture D	**2-person mixture.** 99% one CEU individual, 1% a second CEU individual	Yes	No	Yes
Mixture E	**Complex mixture.** Mixture with 184 individuals at ∼0.2% each, and 41 individuals from Mixture A at ∼1% each.	Yes	No	No
Mixture F	**Complex mixture.** Mixture with 184 individuals at ∼0.2% each, and 47 individuals from Mixture B at ∼1% each.	Yes	No	Yes
Mixture G	**Complex mixture.** Mixture with 184 individuals at ∼0.2% each, and 41 individuals from Mixture B at ∼0.1% each.	No	Yes	No
Mixture H	**Complex mixture.** Mixture with 184 individuals at ∼0.5% each, and 47 individuals from Mixture B at ∼0.1% each.	No	Yes	No

### Mixtures *A1*, *A2*, *B1*, and *B2*: Equimolar Mixtures of HapMap Individuals

Shown in Table 1, two main mixtures (mixtures A and B) were composed in duplicates resulting in a total of 4 mixtures. Mixture A was composed of 41 HapMap CEU individuals (14 trios minus one individual) and mixture B was composed of 47 HapMap CEU individuals (16 trios minus one individual).

### Mixture C1: 90% NA12752 and 10% NA07048

Two CEU males were combined in a single mixture so that one individual (NA12752) contributed 90% (675 ng) of the DNA in the mixture, while the other individual (NA07048) contributed only 10% (75 ng) DNA into the mixture by concentration.

### Mixture C2: 90% NA10839 and 10% NA07048

Two CEU individuals, a female and a male, were combined in a single mixture so that one individual (NA10839) contributed 90% (675 ng) of the DNA in the mixture, while the other individual (NA07048) contributed only 10% (75 ng) DNA into the mixture by concentration.

### Mixture D1: 99% NA12752 and 1% NA07048

Two CEU males were combined in a single mixture so that one individual (NA12752) contributed 99% (742.5 ng) of the DNA in the mixture, while the other individual (NA07048) contributed only 1% (7.5 ng) DNA into the mixture by concentration.

### Mixture D2: 99% NA10839 and 1% NA7048

Two CEU individuals, a female and a male, were combined in a single mixture so that one individual (NA10839) contributed 99% (742.5 ng) of the DNA in the mixture, while the other individual (NA07048) contributed only 1% (7.5 ng) DNA into the mixture by concentration.

### Mixture E: 50% Mixture A1 and 50% Mixture of 184 Equimolar Caucasians

Two mixtures were combined into a single mixture so that each of the original mixtures contributed the same amount of genomic DNA by volume into the final mixture. CAU2 mixture contained 184 Caucasian control individuals obtained from the Coriell Cell Repository. Mixture A1 was constructed as above and contained 41 CEU individuals.

### Mixture F: 50% Mixture B2 and 50% Mixture of 184 Equimolar Caucasians

Two mixtures were combined into a single mixture so that each mixture contributed the same amount of genomic DNA by volume into the final mixture. CAU3 mixture contained 184 Caucasian control individuals obtained from the Coriell Cell Repository. Mixture B2 was constructed as above.

### Mixture G: 5% Mixture A2 and 95% Mixture of 184 Equimolar Caucasians

Two mixtures were combined into a single mixture with Mixture A2 comprising of 5% of the mixture and the CAU3 comprising of 95% of the mixture. CAU3 mixture contained 184 Caucasian control individuals obtained from the Coriell Cell Repository. Mixture A2 was constructed as above.

### Mixture H: 5% Mixture B1 and 95% Mixture of 184 Equimolar Caucasians

Two mixtures were combined into a single mixture with Mixture B1 comprising of 5% of the mixture and the CAU2 comprising of 95% of the mixture. CAU2 mixture contained 184 Caucasian control individuals obtained from the Coriell Cell Repository. Mixture B1 was constructed as above.

### Genotyping

Four cohorts were assayed on the Illumina (San Diego, CA) HumanHap550 Genotyping BeadChip v3, one cohort was assayed on the Illumina (San Diego) HumanHap450S Duo, and three cohorts were assayed on the Affymetrix (Emeryville, CA) Genome-Wide Human SNP 5.0 array, with each cohort being assayed on a single chip. Probe intensity values were extracted for analysis from the file folders generated by the BeadScan software for the Illumina platform, and from Affymetrix GTYPE 4.008 software for the Affymetrix data, as described in previous studies [6].

### Theoretical Derivation of Test-Statistic

We recognize there are multiple approaches to derive a test-statistic to evaluate the hypotheses that a person is within a mixture, and these are discussed further in later sections. In this primary approach we take a frequentist rather than a Bayesian approach, recognizing that both are possible and each has unique advantages.

**Figure 1:**
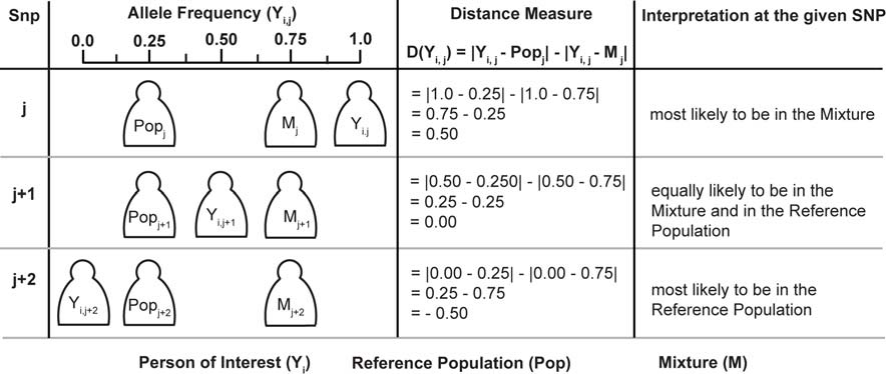
To give insight into the intuition behind our method, we present for a given SNP three different scenarios for the possible allele frequency of the person of interest corresponding to the genotypes AA, AB, and BB. The allele frequencies of the reference population, person of interest, and the mixture are described as *M_i_*, *Y_i_*, and *Pop_i_* respectively. We see that the distance measure is greater (and positive) when the *Y_i_* of the person of interest is closer to the *M_i_* of the mixture than to the *Pop_i_* of the reference population. Similarly, the distance measure is smaller (and negative) when the *Y_i_* of the person of interest is closer to the *Pop_i_* of the reference population than to *M_i_* of the mixture. Our test statistic is then the z-score using this distance measure.

An overview of our approach is described in Figure 1, and this method can be summarized as the cumulative sum of allele shifts over all available SNPs, where the shift's sign is defined by whether the individual of interest is closer to a reference sample or closer to the given mixture. We first introduce our method in terms of genotyping a given SNP for a single person, which addresses the original design of SNP genotyping microarrays for the field of human genetics. We then proceed to adapt our method for mixtures and pooled data.

Current genotyping microarray technology can assay millions of SNPs. Genotypes are expected to result from an assay and data is categorical in nature, e.g. **AA**, **AB**, **BB**, or **NoCall** where **A** and **B** symbolically represent the two alleles for a biallelic SNP. However, as evident from copy number, calling algorithm, and pooling-based GWA studies [6],[17], raw preprocessed data from SNP genotyping arrays is typically in the form of allele intensity measurements that are proportional to the quantity of the “**A**” and “**B**” alleles hybridized to a specific probe (or termed features) on a microarray [16]. Individual probe intensity measurements are derived from the fluorescence measurement of a single bead (e.g. Illumina) or 5 micron square on a flat surface (e.g. Affymetrix). On a genotyping array, multiple probes are present per SNP at either a fixed number of copies (Affymetrix) or a variable number of copies (Illumina). For example, recent generation Affymetrix arrays typically have 3 to 4 probes for the **A** allele and **B** allele respectively, whereas Illumina arrays have a random number of probes averaging approximately 18 probes per allele. With 500,000+ SNPs, there are millions of probes (or features) on a SNP genotyping array. One should note that there are considerably different sample preparation chemistries prior to hybridization between SNP genotyping platforms and thus probes behave differently on the respective platforms.

Before we discuss resolving mixtures, we summarize ‘genotype calling’ in the context of data from a single individual at a single SNP. SNP genotyping algorithms typically begin by transforming normalized data into a ratio or polar coordinates. For simplicity, we will utilize a ratio transformation *Y_i_* = *A_i_*/(*A_i_*+*k_i_B_i_*), where *A_i_* is the probe intensity for the **A** allele and *B* is the probe intensity for the **B** allele for the *j*th SNP. Multiple papers have shown that *Y_j_* transformation approximates allele frequency, where *k_j_* is the SNP specific correction factor accounting for experimental bias and is easily calculated from individual genotyping data [6],[17]. Thus with this transformation, *Y_i_* is an estimate of allele frequency (termed *p_A_*) for each SNP. Since most individuals contain two copies of the genome for autosomal SNPs, values for the **A** allele frequency (*p_A_*) in a single individual may be 0%, 50%, or 100% for the **A** allele at **AA**, **AB**, or **BB**, respectively. Equivocally *Y_i_* will be approximately 0, 0.5, or 1, varying from these values due to measurement noise. By example and assuming *k_j_* = 1, probe intensity measurements of *A_j_* = 450 and *B_j_* = 550 yield *Y_j_* = 0.45 and this SNP would be likely called **AB**. For a single individual, we thus expect to see a trimodal distribution for *Y* across all SNPs since only **AA**, **AB**, or **BB** genotype calls are expected. However, in a mixture of multiple individuals, the assumptions of the genotype-calling algorithm are invalid, since only **AA**, **AB**, **BB**, or **NoCall** are given regardless of the number of pooled chromosomes.

However, this does not prevent us from extracting information and meaning from the relative probe intensity data. In our approach, we compare allele frequency estimates from our mixture (termed *M*, where *M_i_* = *A_i_*/(*A_i_*+*k_i_B_i_*)) to estimates of the mean allele frequencies of a reference population. The selection of the reference population is important and will be discussed later. For now, we assume that the reference population has a similar ancestral make-up as that of the mixture. We refer to having similar population substructure, ethnicity, or ancestral components interchangeably, and define similar ancestral components for an individual or mixture as having similar allele frequencies across all SNPs. We let *Y_i,j_* be the allele frequency estimate for the individual *i* and SNP *j*, where *Y_i,j_*∈{*0*,*0.5*,*1*}, from a SNP genotyping array. We then compare absolute values for two differences. The first difference *|Y_i,j_−M_j_|* measures how the allele frequency of the mixture *M_j_* at SNP *j* differs from the allele frequency of the individual *Y_i,j_* for SNP *j*. The second difference *|Y_i,j_−Pop_j_|* measures how the reference population's allele frequency *Pop_j_* differs from the allele frequency of the individual *Y_i,j_* for each SNP *j*. The values for *Pop_j_* can be determined from an array of equimolar pooled samples or from databases containing genotype data of various populations. Taking the difference between these two differences, we obtain the distance measure used for individual *Y_i_*:
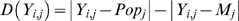
(1)


Under the null hypothesis that the individual is not in the mixture, *D(Y_i,j_)* approaches zero since the mixture and reference population are assumed to have similar allele frequencies due to having similar ancestral components. Under the alternative hypothesis, *D(Y_i,j_)>0* since we expect that the *M_j_* is shifted away from the reference population by *Y_i_*'s contribution to the mixture. In the case of *D(Y_i,j_)<0*, *Y_i_* is more ancestrally similar to the reference population than to the mixture, and thus less likely to be in the mixture. Consistent with the explanation of Figure 1, *D(Y_i,j_)* is positive when *Y_i,j_* is closer to *M_j_* and *D(Y_i,j_)* is negative when *Y_i,j_* is closer to *Pop_j_*. By sampling 500 K+ SNPs, one would generally expect *D(Y_i,j_)* to follow a normal distribution due to the central limit theorem. In our analysis, we take a one-sample t-test for this individual, sampled across all SNPs, and thus obtain the test statistic:
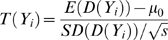
(2)


In equation (2) we assume *μ_0_* is the mean of *D(Y_k_)* over individuals *Y_k_* not in the mixture, *SD(D(Y_i_))* is the standard deviation of *D(Y_i,j_)* for all SNPs *j* and individual *Y_i_*, *and s* is the number of SNPs. We assume *μ_0_* is zero since a random individual *Y_k_* should be equally distant from the mixture and the mixture's reference population and so 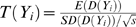. Under the null hypothesis *T(Y_i_)* is zero and under the alternative hypothesis *T(Y_i_)*>*0*. In order to account for subtle differences in ancestry between the individual, mixture, and reference populations among other biases we normalize our allele frequency estimates to a reference population.

### Ancestry and Reference Populations

Different populations will have different mean SNP allele frequencies based on ancestry, admixture, and population bottlenecks. An obvious assumption of this type of analysis is that the reference population must be either (a.) accurately matched in terms of ancestral composition to the mixture and person of interest or (b.) limited to analysis of SNPs with minimal (or known) bias towards ancestry. It is first important to recognize that any single SNP will have only a small effect on the overall test-statistic. Moreover, it is realistic that ancestry of the reference population could be determined by analysis of a small subset of SNPs, followed by analysis of a person's contribution to the mixture with a separate set of SNPs (recognizing that nearly 500,000 SNPs are assayed). In the absence of SNP-specific ancestral information towards allele frequency as was assumed in our study, we can also use normalization methods that leverage the fact that we have assayed hundreds of thousands of SNPs and consequentially have largely sampled the distribution of the test-statistic. In essence, we fit the test-statistic to a second reference population matched to the individual of interest to account for ancestry differences that do not effect the overall distribution of allele frequencies. Thus under the assumption of similar test-statistic distributions, normalizing SNP data from the mixture to a reference population reduces the effect of systematic biases on allele frequency from the microarray or, to an extent, towards ancestry at a cost of power.

While not necessary in this study, the effect of ancestry on allele frequency could be more directly managed by SNP selection combined with extensive allele frequency data across multiple ancestrally diverse populations. Ideally, one would use a subset of SNPs to identify ancestry of the individual and then match them to a reference population. Moreover, SNPs that are stable for allele frequency across populations (low *F_st_*) or at have a common distribution of allele frequencies would be preferable. Identifying such a set of SNPs and more appropriately considering ancestral biases are reserved for future database studies whereby genotype data of an ancestrally diverse set of individuals is available.

### Software

Pre-compiled UNIX binaries are available for a software implementation of our method and can be found at http://public.tgen.org/dcraig/deciphia. Our software is able to run analysis using raw data from either Affymetrix or Illumina or by using genotype calls. The software is also able to normalize our test statistic using the reference population and/or adjust the mean test statistic using a specified individual. Additionally, the user can restrict the SNPs considered to a subset of the total available SNPs. For raw input data we are able to match the distribution of signal intensities for each raw data file to that of the mixture input file (see platform specific analysis). Finally, multiple test statistics and distance calculations are implemented including our original test statistic, Pearson correlation, Spearman rank correlation and Wilcoxon sign test.

### Platform Specific Analysis

With the Affymetrix platform we were able to use genotypes for each individual and found similar results with the Illumina platform. Additionally, we were able to use the raw CEL files from the HapMap dataset [18] found at http://www.HapMap.org. To overcome the differences in distribution of signal intensity between CEL files, we matched the distribution of the signal intensities to the distribution of the mixture's CEL file. This was achieved by ordering allele frequencies on a given chip (and allele frequencies in the mixture). We then substituted the *i* ^th^ allele frequencies from the mixture of interest for the *i* ^th^ allele frequencies for the given chip. Without this adjustment, there was difficulty resolving any individual in any mixture due to the fact that we did not account for off-target cross-hybridization. This type of adjustment is the preferred type of normalization method when raw data is available for the mixture, person of interest, and reference population.

For the Illumina platform we used the genotypes from the HapMap dataset [18] for both the person of interest and the reference populations instead of raw intensity values as we had for the Affymetrix platform. For the mixture we used raw intensity values. This set of data mimics the case where raw data may not be available but genotype calls are available. We use a simple method to reduce errors between different microarrays, where we normalize each microarray by dividing by the mean channel intensity for each respective channel. This was performed on the raw data for the mixture only. We note that this platform specific adjustment is not needed when the raw data for a person's genotype is present on the same platform. In the Illumina specific example, we utilized only the calls from the HapMap without having platform specific genotype data. Theoretically, it should be possible to use a library of *Y_i_* means for **AA**, **AB**, and **BB** to map genotype calls to expected *Y_i_* values to each SNP for individually genotyped samples, but this was not necessary for our analysis.

### Simulation

Simulation was used to test the efficacy of using high-density SNP genotyping data for resolving mixtures. The key variables of the simulation are: the number of SNPs *s*, the fraction *f* of the total DNA mixture contributed by our person of interest *Y_i_*, and the variance or noise inherent to assay probes *v_p_*. In the simulations, theoretical mixtures were composed by randomly sampling individuals from the 58C Wellcome Trust Case-Control Consortium (WTCCC) dataset [19]. After removing duplicates, relatives and other data anomalies, a total of 1423 individuals remained for sampling. The genotype calls for these individuals were provided from the WTCCC and were previously genotyped on the Affymetrix 500 K platform. Within each simulation, we randomly chose *N* individuals to be equally represented in our mixture and then computed the mean allele frequency (*Y_i_*) of our mixture for each SNP. SNPs *j* with an observed *Y_ij_* below 0.05 or above 0.95 in the reference population were removed due to their potential for having false positives and low inherent information content.

We then simulated a microarray that would contain a mean of 16 probes for simplicity, approximating the mean number of probes found on the Illumina 550 K, Illumina 450S Duo and Affymetrix 5.0 platforms (18.5, 14.5 and 4 respectively). For each SNP *j* we added to the *Y_ij_* of each probe a Gaussian noise based off the previously measured probe variance. When fixed, we set probe variance to 0.006 when simulating Affymetrix 5.0 arrays, and to 0.001 for both Illumina 550 K and Illumina 450S Duo arrays. The allele frequency of the mixture was then calculated to be the mean of these probe values. A mixture size of *N* is equivalent to saying that an individual's DNA represents *f* = *1/N^th^* of the total DNA in the mixture. We tested equimolar mixtures ranging from 10 individuals to 1,000 individuals. Using this design, we tested each individual for their presence where they contributed between 10% and 0.1% genomic DNA to the total mixture. To obtain significance levels (p-values) for testing the null hypothesis, we sampled from the normal distribution. We note that we do not have enough samples to test the tail of our distribution and therefore our p-values are not completely accurate (e.g. below 10^−6^). Nonetheless, p-values are expected to be sufficiently accurate to qualitatively assess the limits of our method.

### Experimental Validation

To examine empirically the efficacy of our method we formed various known mixtures of DNA from HapMap individuals and genotyped the mixtures on three different platforms. Listed in Table 1 and detailed in the methods are the compositions of the different mixtures formed and the platforms they were assayed across. The use of mixtures of HapMap individuals has several advantages. First, we can be confident of the genotype calls because in most cases more than one platform has been used to identify the consensus genotype. Second, trios are available, which allow for evaluation of identifying an individual using a relative's genotype data. Third, by using mixtures of multiple HapMap individuals we can evaluate our ability to resolve each individual within the mixture. Therefore we have constructed simple two-person mixtures as well as complex mixtures containing contributions from 40+ individuals. For each mixture, we used the HapMap CEU individuals not present in the mixture as our reference population for the mixture.

## Results

Using the theoretical framework established in the methods, we evaluated the feasibility of using high-density SNP genotyping data to resolve complex mixtures. First, we constructed a series of simulations to evaluate the theoretical limits of resolving an individual within a mixture using the described analytical framework and given characteristics of current generation SNP genotyping microarrays. Second, we experimentally tested the feasibility of detecting if an individual is contributing trace amounts of DNA to highly complex mixtures. Within these simulations and experimental tests, particular focus was given on complex mixtures–those containing hundreds or thousands of individuals. While these mixtures are more complex than those of previous studies, they can be used to evaluate the theoretical bounds of current technology and to justify the use of reduced platforms for practical application. Conceptually, such approaches may have utility in resolving a mixture of DNA from common surfaces where many individuals have left DNA.

### Simulation

We performed simulations to examine the trade-off between the number of SNPs considered, the fraction of the DNA mixture belonging to our person of interest, and the probe variance or noise of the microarray.

#### Joint Adjustment of Mixture Fraction (f) and Number of SNPs (s)

**Figure 2:**
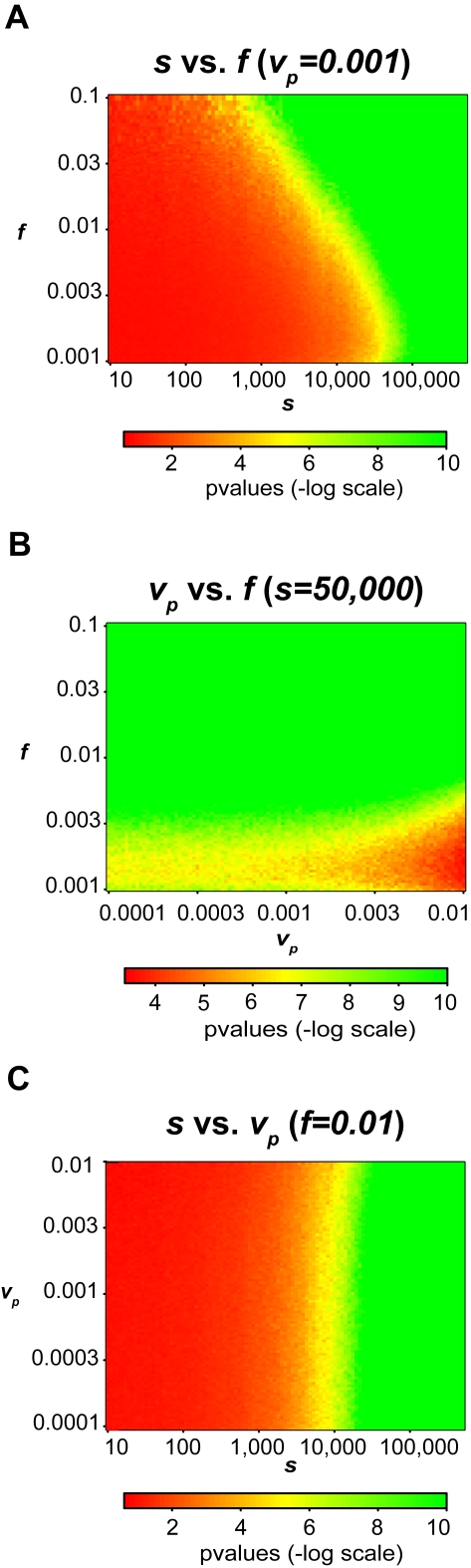
Simulation Results. Using 1423 Wellcome Trust 58C individuals, we give log scaled p-values for simulations based on three variables: the number of SNPs (*s*), the fraction of the individual in the mixture (*f*), and the probe variance (*v_p_*). The graphs plot the relationships between the three variables with a different variable fixed in each graph. The log scaled p-values are represented by the color of each point in the graph, as well as the z-axis on the right graphs. These simulations suggest that we should be able to resolve mixtures where a given individual is 0.1% of the mixture (*f*), probe variance is at most 0.01 (*v_p_*) and the number of SNPs probed is 50,000 (*s*).

We first examined the trade-off between the numbers of SNPs considered versus the fraction of the DNA mixture belonging to our person of interest. Clearly, we expect greater ability to resolve individuals from a mixture when more SNPs are used in the calculation, though the absolute limits of detection are ultimately determined by the genetic variation of the population. We assumed a variance (*v_p_*) for the estimated allele frequency of each probe of 0.001, which follows closely our observed variance (0.00158) of the Illumina 550 K platform across multiple arrays in other genotyping studies. Figure 2a shows 10,000 simulations ranging from *s* = *10* to *s* = *500,000* and *f* = *0.1* to *f* = *0.001*, where the Z-axis is the p-value. We see that with only 10,000 to 25,000 SNPs we were able to resolve mixtures where the person of interest was less than 1% of the total mixture at a p-value of less than 10^−6^. To resolve mixtures where the person of interest is less than 1% of the total mixture, conservatively 25,000 SNPs are required to achieve a p-value of less than 10^−6^. At the extreme, if we use all the available SNPs, we can easily resolve mixtures where our person of interest is less than 0.1% of the total mixture to achieve a p-value of less than 10^−6^.

#### Joint Adjustment of Probe Variance (v_p_) and Mixture Fraction (f)

In these simulations, we assume that we have 50,000 SNPs on each microarray (*s* = 50,000). While conceivably we could use a much greater number of SNPs, the lower number of SNPs would be more realistic in a setting where preference has been given to SNPs whose allele frequencies minimally vary across different populations. Figure 2b shows 10,000 simulations from *v_p_* = *0.0001* to *v_p_* = *0.01* and *f* = *0.1* to *f* = *0.001*. We see that within a small amount of probe variance we are able resolve an individual who comprises of one-thousandth of a mixture. If the probe variance is below 0.001 we are able to easily resolve an individual whose DNA comprises 10% to 0.1% of the mixture. Even with increasing noise, we are still able to resolve mixtures where the person of interest contributes less than 2.5% with a p-value of less than 10^−6^. We also observe that the probe variance does not have a large impact on the p-value, and in this case the fraction of the mixture is the important factor when the number of SNPs is fixed.

#### Joint Adjustment of Number of SNPs (s) and Probe Variance (v_p_)

Finally we examined the trade-off between the number of SNPs and the probe variance. We assume that our person of interest contributes 1% to the mixture (*f* = *0.01*). Figure 2c shows 10,000 simulations from *s* = *10* to *s* = *500,000* and *v_p_* = *0.0001* to *v_p_* = *0.01*. The probe variance has little effect on the significance of the test. Consequently, it would be sufficient to use 50,000 SNPs, even with very high levels of noise to resolve mixtures of sizes up to 100. We note that within simulations the number of probes is fixed to be 16, and thus the noise does not affect the allele frequency estimate, as would be the case with arrays using 4 probes.

#### Equimolar Mixtures versus Two-Person Mixtures

We performed the same three simulation designs for mixtures that only included two individuals. Instead of *N* = *1/f* individuals contributing equally to the mixture, we create mixtures where individual one would make up *(N−1)/N* of the mixture and individual two would make up *1/N* of the mixture. When the three simulations were performed we observed an increase in significance (smaller p-values). This gives further utility to the method when there are a small number of total contributors with the person of interest making up only a small fraction of the mixture.

#### Conclusions from Simulations

Our simulations demonstrate that 10,000 to 50,000 SNPs can resolve mixtures where the genomic DNA of the person of interest composes 10% to 0.1% of the DNA within the total mixture. Noise plays an important but secondary role since microarray technologies such as the Illumina 550 K and Illumina 450S Duo platforms have a sufficiently large number of replicate probes compared to population sampling variance. Another consideration is that the choice of SNPs was not made with any specific intent and therefore we could potentially reduce the number of SNPs significantly if we choose the most informative SNPs, for example by choosing a set of SNPs that do not vary across differing populations.

### Experimental Validation

To examine empirically the efficacy of our method we formed various known mixtures of DNA from HapMap individuals and genotyped the mixtures on three different platforms.

#### Resolving an Individual within Mixtures of 40+ Individuals

**Figure 3:**
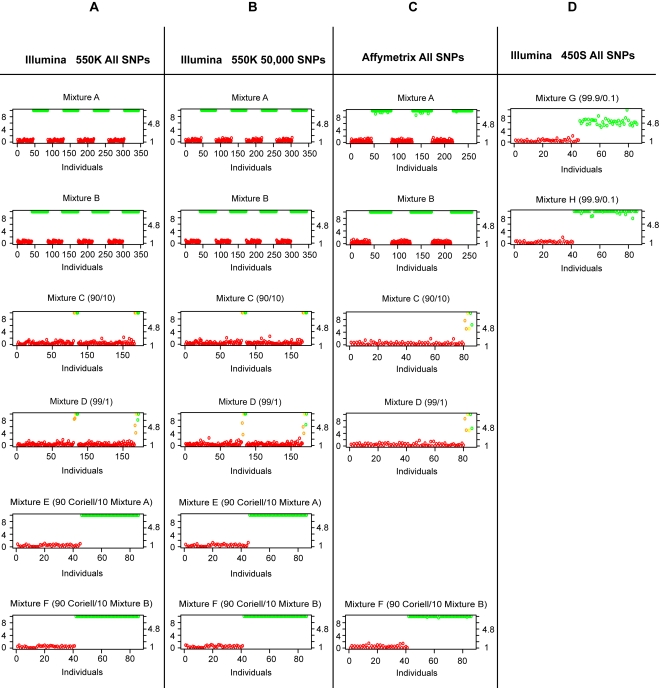
Experimental validation using a series of mixtures (see Methods A–F) assayed on the Affymetrix GeneChip 5.0, Illumina BeadArray 550 and the Illumina 450S Duo Human BeadChip. The x-axis shows each individual in the CEU HapMap population, the left y-axis shows the p-value (log scaled), and the right y-axis shows the value of the test statistic. For mixtures A, B, E and F those in the mixture are colored green and those not in the mixture are colored red. For mixtures C and D those individuals who are not in the mixtures are colored red, those individuals who are related to the 1% or 10% individuals in the mixtures are colored orange, those individuals who are related to the 90% or 99% are colored yellow, and those people in the mixture are colored green. In all mixtures, the identification of the presence of a person's genomic DNA was possible.

Figure 3 shows the test-statistic for each individual within each mixture. Both individuals in the mixture and not in the mixture were tested for presence within the mixture. On each graph, the left y-axis represents the −log p-value, the right y-axis represents the normalized test-statistic *S(Y_i,j_)*, and the bottom axis represents each individual. We performed each experiment more than once and thus we have multiples of 86 individuals indexed on the bottom axis. For mixtures A, B, E, F, G and H, those in the mixture are colored green and those not in the mixture are colored red. All individuals in the mixtures composed of more than 40 individuals were identified with zero false positives.

#### Resolving Members within 2 Person Mixtures (f = 1% and f = 10%)

For mixtures C and D, those individuals who are not in the mixtures are colored red, those individuals who are related to a person in the mixture are colored orange, and those people in the mixture are colored green. We were able to correctly identify individuals within the mixture with zero false-positives except, as expected, for relatives of individuals in the mixture, which appear at a midpoint between those in and those not in the mixture.

#### Resolving an Individual from a Mixture using a Relative's Genotypes

It is interesting to observe that we have no false-positives in the Mixture A, B, E, F, G or H but we do have false-positives when considering Mixture C and D. This is not unexpected since the HapMap CEU population is composed of trios and we are in fact resolving that the mother or father of the individual (a son or daughter) is in the mixture; the yellow and orange marked individuals being observed as significant in Figures 3a and 3c. Thus, we can easily resolve an individual (son or daughter) even when using their mother's or father's genotypes.

#### Resolving an Individual from a Mixture with 50,000 SNPs

In Figure 3a, we see that all the mixtures are able to be resolved with no false-negatives when we use all 504,605 SNPs present on the Illumina 550 K platform. We performed the same analysis considering only 50,000 SNPs (see Figure 3b) and found that the samples had the same degree of separation. Thus, even if only a small fraction of the intended genotypes are generated (such as in a degraded sample), identification of an individual in a complex mixture is possible.

#### Resolving an Individual when Contributing Less than 1%

In Figure 3d, we consider mixtures G and H where the fraction of DNA of each individual is between 0.15% and 0.25% of the total mixture. We see that using all the SNPs available we are able to resolve all the mixtures with no false-negatives on the Illumina 450S Duo platform. We can therefore resolve an individual even when the fraction of their DNA in the mixture is less than 1%.

## Discussion

Within this study, we develop a theoretical framework for resolving mixtures using high-density SNP array data, use simulation to test the limitations of these approaches, and experimentally demonstrate rapid and robust determination of whether individuals are within an assayed mixture. Our results show a remarkable ability to identify trace amounts of an individual's DNA within highly complex mixtures. These results further suggest novel forensic applications where the existence of DNA from numerous other individuals currently hampers the ability to identify the presence of any single individual.

Whereas few conclusions can be drawn by a SNP measurement that is slightly biased (less than 1%) towards an individual's genotype, considerable confidence is gained by statistical analysis of the cumulative aggregate of all measurements across millions of SNPs. While in hindsight this conclusion seems obvious, it represents a fundamental paradigm shift in thinking about the utility of SNPs at resolving mixtures. The approach described here uses the ratio of intensity measures from common biallelic SNPs. As a result, one expects more robust scaling in DNA quantity or quality at any given SNP. We assume neither a known number of individuals present in the mixture nor the presence of equal amounts of DNA from each individual within the mixture. Described in simplistic terms, we determine whether a person is in a mixture by comparing a statistically describable distance measure between the individual and the mixture versus the individual and the reference population.

The analytical framework presented within this study builds upon pioneering approaches for assessing and quantifiably calculating whether a person is within a mixture. These methods have frequently employed match probability estimation after inferring genotypes using STRs, where the probability of two unrelated individuals sharing a combination of markers is calculated [8]. Exclusion probabilities give a calculation based on the probability of excluding a random individual [20]. Nevertheless, many of these methods rely on assuming the number of individuals in the mixture [1] (which is not necessary in our analysis) and have been applied only to STR markers.

One can also consider using other statistical approaches. For example, likelihood ratios are also commonly used when testing which hypothesis is favored by DNA evidence [21]. Adapting to the overall framework presented in this study, one might compute the likelihood ratio of two hypotheses: the individual contributes to the mixture and the individual does not contribute to the mixture. The proper prior odds ratio can then be given based on the current situation or context, and then would be combined with the likelihood ratio to give a posterior odds ratio. In this approach, using SNP microarrays for allele frequencies or allele counts could be used to calculate the probability of the observed mixture's allele frequency or individual of interest's genotype. This Bayesian approach could build from the methods presented here and, depending on the scenario, has attractive strengths including creation of explicit hypotheses (e.g. that a person and/or related individuals are within the mixture) and inclusion of specific priors (e.g. informativeness towards ancestry SNPs). Overall, it is clear there are multiple analytical methods for resolving complex mixtures and depending on the objective, other methods may be more suitable. Regardless of method, it is clear that the perception that SNPs cannot be easily used to resolve mixtures is no longer valid.

Given the results of this study, it is possible to speculate on future research assessing the viability of using commonly handled surfaces as a forensics source. In the context of degraded samples, further research will be needed to choose which SNPs (of millions assayed SNPs) provide sufficient amplifiable DNA or show less allelic bias at low concentrations. Further, the theoretical principles described here will apply to mitochondrial variants. Regardless of the artifacts encountered, the large number of assayed SNPs may allow for partitioning sets of SNPs for different analyses, such that a small subset of SNPs becomes reserved for detecting specific artifacts, such as biases in allele amplification or ancestry. Additional areas of future research include conversion tables using haplotype or imputation frameworks to convert between SNPs and microsatellite markers.

Finally, it is important to consider these findings in light of GWA studies. Indeed, the push to develop high-density SNP genotyping arrays is largely driven by the desire to identify common variants predisposing to a disease. For many GWA studies, the overall cost of genotyping thousands of individuals is substantial. However since genotype data is transferable and can be combined with data from other studies, there is a considerable effort to make experimental data publicly available. As part of this effort, many studies provide pooled allele frequency data in the form of summary statistics (e.g. allele frequencies or genotype counts), in part to mask individual-level genotype data. Though counter-intuitive, our findings show a clear path for identifying whether specific individuals are within a study based on summary-level statistics. Such approaches may have specific utility for identifying redundant individuals when new individual-level genotype data is combined with previous studies sharing only summary statistics.

Considering privacy issues with genetic data, it is now clear that further research is needed to determine how to best share data while fully masking identity of individual participants. However, since sharing only summary data does not completely mask identity, greater emphasis is needed for providing mechanisms to confidentially share and combine individual genotype data across studies, allowing for more robust meta-analysis such as for gene-environment and gene-gene interactions.

## References

[1] Egeland T, Dalen I, Mostad PF (2003). Estimating the number of contributors to a DNA profile.. Int J Legal Med.

[2] Hu YQ, Fung WK (2003). Interpreting DNA mixtures with the presence of relatives.. Int J Legal Med.

[3] Balding DJ (2003). Likelihood-based inference for genetic correlation coefficients.. Theor Popul Biol.

[4] Clayton TM, Whitaker JP, Sparkes R, Gill P (1998). Analysis and interpretation of mixed forensic stains using DNA STR profiling.. Forensic Sci Int.

[5] Cowell RG, Lauritzen SL, Mortera J (2007). Identification and separation of DNA mixtures using peak area information.. Forensic Sci Int.

[6] Pearson JV, Huentelman MJ, Halperin RF, Tembe WD, Melquist S (2007). Identification of the genetic basis for complex disorders by use of pooling-based genomewide single-nucleotide-polymorphism association studies.. Am J Hum Genet.

[7] Bill M, Gill P, Curran J, Clayton T, Pinchin R (2005). PENDULUM–a guideline-based approach to the interpretation of STR mixtures.. Forensic Sci Int.

[8] Jobling MA, Gill P (2004). Encoded evidence: DNA in forensic analysis.. Nat Rev Genet.

[9] Ladd C, Lee HC, Yang N, Bieber FR (2001). Interpretation of complex forensic DNA mixtures.. Croat Med J.

[10] Goodwin W, Linacre A, Vanezis P (1999). The use of mitochondrial DNA and short tandem repeat typing in the identification of air crash victims.. Electrophoresis.

[11] Coble MD, Just RS, O'Callaghan JE, Letmanyi IH, Peterson CT (2004). Single nucleotide polymorphisms over the entire mtDNA genome that increase the power of forensic testing in Caucasians.. Int J Legal Med.

[12] Parsons TJ, Coble MD (2001). Increasing the forensic discrimination of mitochondrial DNA testing through analysis of the entire mitochondrial DNA genome.. Croat Med J.

[13] Just RS, Irwin JA, O'Callaghan JE, Saunier JL, Coble MD (2004). Toward increased utility of mtDNA in forensic identifications.. Forensic Sci Int.

[14] Vallone PM, Just RS, Coble MD, Butler JM, Parsons TJ (2004). A multiplex allele-specific primer extension assay for forensically informative SNPs distributed throughout the mitochondrial genome.. Int J Legal Med.

[15] Kidd KK, Pakstis AJ, Speed WC, Grigorenko EL, Kajuna SL (2006). Developing a SNP panel for forensic identification of individuals.. Forensic Sci Int.

[16] Kennedy GC, Matsuzaki H, Dong S, Liu WM, Huang J (2003). Large-scale genotyping of complex DNA.. Nat Biotechnol.

[17] Macgregor S, Zhao ZZ, Henders A, Nicholas MG, Montgomery GW (2008). Highly cost-efficient genome-wide association studies using DNA pools and dense SNP arrays.. Nucleic Acids Res.

[18] (2003). The International HapMap Project.. Nature.

[19] (2007). Genome-wide association study of 14,000 cases of seven common diseases and 3,000 shared controls.. Nature.

[20] Chakraborty R, Meagher TR, Smouse PE (1988). Parentage analysis with genetic markers in natural populations. I. The expected proportion of offspring with unambiguous paternity.. Genetics.

[21] Weir BS, Triggs CM, Starling L, Stowell LI, Walsh KA (1997). Interpreting DNA mixtures.. J Forensic Sci.

